# Risk of Anxiety and Depression after Diagnosis of Young-Onset Colorectal Cancer: A Population-Based Cohort Study

**DOI:** 10.3390/curroncol29050249

**Published:** 2022-04-27

**Authors:** Alyssa Howren, Eric C. Sayre, Vicki Cheng, Niki Oveisi, Helen McTaggart-Cowan, Stuart Peacock, Mary A. De Vera

**Affiliations:** 1 Faculty of Pharmaceutical Sciences, University of British Columbia, Vancouver, BC V6T 1Z3, Canada; alyssa.howren@ubc.ca (A.H.); vcheng1@student.ubc.ca (V.C.); niki.oveisi@ubc.ca (N.O.); 2 Collaboration for Outcomes Research and Evaluation, Vancouver, BC V6T 1Z3, Canada; 3 Arthritis Research Canada, Vancouver, BC V5Y 3P2, Canada; esayre@arthritisresearch.ca; 4 BC Cancer, Vancouver, BC V5Z 4E6, Canada; hcowan@bccrc.ca (H.M.-C.); speacock@bccrc.ca (S.P.); 5 Faculty of Health Sciences, Simon Fraser University, Burnaby, BC V5A 1S6, Canada; 6 Centre for Health Evaluation and Outcome Sciences, Vancouver, BC V6Z IY6, Canada

**Keywords:** colorectal cancer, depression, anxiety

## Abstract

Given the increasing incidence of young-onset colorectal cancer (yCRC; <50 years), we aimed to evaluate the risk of depression and anxiety in individuals with yCRC in comparison to average-age-onset CRC (aCRC; ≥50 years) and to cancer-free controls, with stratification by sex. Our cohort study identified individuals (≥18 years) with CRC and cancer-free controls (10:1) matched on age and sex using population-based linked administrative health databases in British Columbia, Canada. We assessed depression and anxiety using validated algorithms. We evaluated the risk of depression and anxiety using multivariable Cox proportional hazard models. The cohort included 54,634 individuals with CRC (46.5% female, mean age 67.9 years) and 546,340 controls (46.5% female, mean age 67.9 years). Those with yCRC as compared to aCRC had an increased risk for depression (adjusted hazard ratio [aHR] 1.41; 95% confidence interval [CI] 1.25 to 1.60), and when stratified by sex, the risk was only significant among males (aHR 1.76; 95% CI 1.48 to 2.10). When comparing individuals with yCRC to cancer-free controls, the overall risk of depression (aHR 1.00; 95% CI 0.92 to 1.10) and anxiety (aHR 1.10; 95% CI 0.95 to 1.27) was non-significant; however, males had a significantly higher risk for mental health disorders, specifically depression (aHR 1.17; 95% CI 1.03 to 1.33). Altogether, our findings that individuals with yCRC experience higher risk of depression compared to those with aCRC as well as cancer-free controls, particularly among males, suggest effects of age and sex on mental health outcomes.

## 1. Introduction

In Canada, colorectal cancer (CRC) accounted for 11% of all new cancer cases in 2021, and in 2019 was the third most commonly diagnosed cancer among both males (12.9%) and females (10.9%) [1,2]. CRC is traditionally considered a disease in older adults, with average-age-onset CRC (aCRC) occurring in individuals 50 years or older. However, research over the past decade has shown that incidence of young-onset colorectal cancer (yCRC), among individuals younger than 50 years, has increased steadily. A 2019 Canadian study reported yCRC incidence among males and females increased by a mean annual percentage change of 3.47% from 2006 to 2015, and 4.45% from 2010 to 2015, respectively [3].

The increasing incidence of yCRC has called for psycho-oncology research on psychological, social, and behavioural impacts on patients. Of particular interest are mental health impacts of yCRC, as previous research has shown that CRC patients are at a higher risk of mental health disorders compared to the general population [4]. A 2017 cohort study using the Taiwanese National Health Insurance Research Database found that CRC patients had increased risk of developing mood disorders (adjusted hazard ratio [aHR] 3.05; 95% confidence interval [CI] 2.89 to 3.20) with higher risk in those diagnosed with CRC less than or at 49 years of age (aHR 3.75; 95% CI 3.28 to 4.29), compared to general population controls [5]. A 2019 US cohort study using the Utah Population Database showed that CRC patients were at an increased risk for any mental health disorders after diagnosis at 0–2 years (aHR 3.70; 95% CI 3.47 to 3.95), >2–5 years (aHR 1.23; 95% CI 1.09 to 1.38), and ≥5 years (aHR 1.20; 95% CI 1.07 to 1.36) [6]. Although authors evaluated potential effects of age at diagnosis, they used a cut-off of 65 years, which preclude the ability to extrapolate findings to those with yCRC. Additionally, a 2021 US study evaluated the onset of newly diagnosed depression after CRC diagnosis and showed positive association among males (odds ratio [OR] 1.89; 95% CI 1.85 to 1.93) and negative association among females (OR 0.53; 95% CI 0.52 to 0.54) [7]. A similar cut-off of 65 years limits the ability to understand findings in the context of yCRC, while findings on sex differences warrant further investigation. Furthermore, the lack of validated case definitions for mental health outcomes in the aforementioned studies may be associated with biased estimates with the unknown magnitudes of risk. To address these limitations with a focus on yCRC, our objectives are: (1) to evaluate the risk of mental health disorders, namely anxiety and depression, among individuals with yCRC compared to aCRC; and (2) to evaluate the risk of mental health disorders among individuals with yCRC, aCRC, and CRC overall and respective cancer-free controls. Evaluations were informed by a sex-based analysis approach, which is relevant as current literature on CRC outcomes suggests differences between males and females, and mental health outcomes are influenced by patients’ biological and social contexts [8,9,10,11].

## 2. Materials and Methods

### 2.1. Data Source and Study Population

We used linked administrative health databases capturing longitudinal and deidentified individual-level health services data for the province of British Columbia (BC), Canada (approximately 4.86 million in 2016 [12]) [13,14,15,16,17,18,19]. Population Data BC facilitated data access to health databases from April 1985 onwards, which include the Medical Services Plan database on outpatient visits [18], the Discharge Abstract Database on inpatient visits [19], the Consolidation File for demographics [16], and Vital Statistics File for deaths [17]. These databases were linked to the BC Cancer Registry, which captures information on cancers diagnosed in people living in BC from 1985 onwards [14]. The BC Cancer Registry includes data on cancer diagnosis (e.g., date, tumour group, sites, stage) and treatment (e.g., dates, modality).

**Figure 1:**
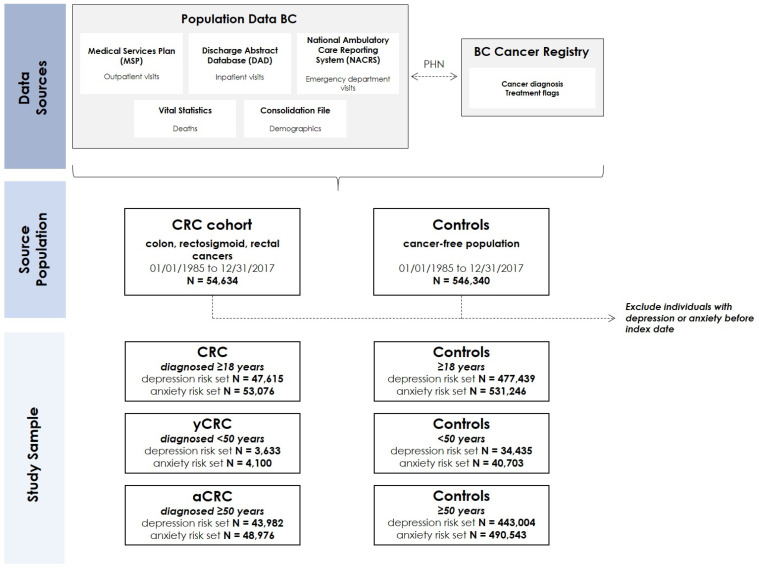
Overview of data source, source population and study sample (dashed arrows show linkages between databases using personal health numbers which are then de-identified/scrambled). *The risk set refers to individuals without a history of depression or anxiety and are therefore considered at risk for incident depression or anxiety and included in the study sample for each respective analysis. Abbreviations: CRC—colorectal cancer; yCRC—young-onset colorectal cancer; aCRC—average-age-onset colorectal cancer; PHN—personal health number*.

The study population was comprised of individuals diagnosed with CRC from the period of 1 January 1985 to 31 December 2017. We identified individuals with CRC through the BC Cancer Registry using *International Classification of Diseases for Oncology, Third Edition* codes, specifically: C18.2–C18.9 (colon); C19.9 (rectosigmoid); and C20, C21.8 (rectum). Individuals with CRC were matched to cancer-free controls (1:10) based on age, sex, and index date, resulting in a study population of 54,634 individuals with CRC and 546,340 controls. We further classified individuals as yCRC, defined as individuals who received their diagnosis at less than 50 years of age and aCRC for individuals who received their diagnosis at 50 years of age or greater (see Figure 1 for data sources and source population). We determined age of CRC diagnosis using date of birth from Population Data BC and the date of diagnosis from the BC Cancer Registry.

### 2.2. Outcome Ascertainment

The primary outcomes were incident depression and anxiety which were ascertained with validated case definitions that use both outpatient (from Medical Services Plan database) and inpatient (from Discharge Abstract Database) data [20,21,22]. The specific *International Classification of Diseases (ICD)* codes were: depression—ICD-9: 296.2, 296.3, 296.5, 300.4, 309.x, and 311.x; ICD-10: F20.4, F31.3-F31.5, F32.x, F33.x, F34.1, F41.2, and F43.2; and anxiety—ICD-9: 300.0, 300.2; ICD-10: F40-F41. The case definition for depression was operationalized as: (1) ≥1 inpatient ICD code; or (2) 2 outpatient ICD codes within a 1-year period [20]. To meet the case definition for anxiety, the aforementioned ICD codes were operationalized as: (1) ≥1 inpatient ICD code; or (2) 2 outpatient ICD codes within a 2-year period [21,22]. Individuals with CRC or cancer-free controls that met the case definition for depression or anxiety before the index date, defined as the date of CRC diagnosis or matched date for controls, were excluded to ensure identification of incident cases of depression or anxiety (Figure 1).

### 2.3. Covariate Ascertainment

A key covariate in our analysis is sex, which represents the biological construct, and recorded in the Consolidation File. Administrative databases in BC do not yet capture information on the social construct, gender. Other covariates evaluated in our analysis included age, residence (urban/rural), and neighbourhood income quintile. We also evaluated medical comorbidities using the Charlson–Romano comorbidity index [23] and health care use according to the annual frequency of outpatient and inpatient visits. All covariates were assessed in the year prior to index date. Additionally, for the period of 1 January 2010 to 31 December 2017, we included data on cancer stage (i.e., stage 0/1, 2, 3, and 4). This corresponded to population-based reporting of staging data, based on American Joint Committee on Cancer staging guidelines, which began in 2010, with >85% capture in the BC Cancer Registry [24,25].

### 2.4. Statistical Analysis

Descriptive statistics were determined for all incident cases of CRC, yCRC, and aCRC and cancer-free controls in the year prior to index date. Our primary analyses evaluated the risk of depression and anxiety among individuals with yCRC in comparison to aCRC using multivariable Cox proportional hazard regression models, adjusted for age, sex, neighbourhood income quintile, residence, Charlson–Romano comorbidity index, and health care use. This primary analysis was repeated within the restricted calendar period of 1 January 2010 to 31 December 2017 to adjust for the stage of cancer at diagnosis. In the secondary analysis, we evaluated the risk of depression and anxiety among individuals with CRC, yCRC, and aCRC in comparison to controls using multivariable Cox proportional hazard regression models that adjusted for age, sex, neighbourhood income quintile, residence, Charlson–Romano comorbidity index, and health care use. To consider sex, primary and secondary analyses were repeated among females and males. Lastly, we assessed when in the trajectory of CRC care that individuals were being diagnosed with depression and anxiety. Specific phases of CRC care that we evaluated were: (1) initial (12 months after CRC diagnosis); (2) continuing (time between initial and end-of-life phases); and (3) end-of-life (12 months before cancer death) [26]. We completed all of these analyses using SAS statistical software v. 9.4 (SAS Institute, Cary, NC, USA).

### 2.5. Study Conduct

This study was approved by the University of British Columbia (H17-03530). All inferences, opinions, and conclusions drawn in this manuscript are those of the authors, and do not reflect the opinions or policies of the Data Steward (s).

## 3. Results

Altogether, the study population included 54,634 individuals with CRC (46.5% female, mean age 67.9 years); of which, 4223 had yCRC (49.5% female, mean age 43.2 years) and 50,411 were diagnosed with aCRC (46.2% female, mean age 70.0 years). Descriptive characteristics of individuals with CRC, yCRC, and aCRC, along with their respective cancer-free controls, are summarized in Table 1.

### 3.1. Risk of Depression and Anxiety for yCRC in Comparison to aCRC

The incidence rate of depression among individuals with yCRC was 19.2 per 1000 person years and for aCRC was 17.1 per 1000 person years. For anxiety, the incidence rate for individuals with yCRC was 5.9 per 1000 person years and among individuals with aCRC was 4.6 per 1000 person years. The unadjusted hazard ratios (HR) for depression and anxiety for individuals with yCRC compared to aCRC were 1.15 (95% CI 1.05 to 1.25) and 1.30 (95% CI 1.12 to 1.50), respectively. Multivariable models that adjusted for the confounding effects of age, sex, comorbidity index, neighbourhood income quintile, and health care use resulted in an attenuated and non-significant HR for anxiety (aHR 1.05; 95% CI 0.86 to 1.28). In contrast, the aHR ratio for depression among individuals with yCRC compared to aCRC was increased and significant at 1.41 (95% CI 1.25 to 1.60). When these multivariable models were stratified by sex, the risk of depression among males with yCRC compared to those with aCRC remained as the only significant association, with an aHR of 1.76 (95% CI 1.48 to 2.10) (Table 2).

The above analyses were repeated for the restricted calendar period of 1 January 2010 to 31 December 2017 when cancer staging data were available for multivariable adjustment (Table 3). Findings on the risk of incident depression and anxiety for those with yCRC compared to aCRC were similar to analyses without cancer stage adjustment, as the aHR for depression was 1.56 (95% CI 1.13 to 2.14) and risk of anxiety was non-significant (aHR 1.17; 95% CI 0.79 to 1.75). Stratifying the multivariable models according to sex showed that males with yCRC relative to males with aCRC had an increased risk of incident depression (aHR 1.60; 95% CI 1.02 to 2.52).

### 3.2. Risk of Depression and Anxiety for CRC, yCRC and aCRC in Comparison to Controls

The incidence rate of depression among individuals with CRC was 17.3 per 1000 person years and for cancer-free controls was 15.4 per 1000 person years. Fewer individuals experienced anxiety, as incidence rates among individuals with CRC was 4.8 per 1000 person years and was 4.0 per 1000 person years for cancer-free controls. Presented in Table 4 are unadjusted and adjusted HRs for the risk of depression and anxiety in individuals with CRC, yCRC, and aCRC as compared to their respective controls. Overall, the aHRs for depression and anxiety in individuals with CRC as compared to cancer-free controls were 1.03 (95% CI 1.00 to 1.06) and 1.11 (95% CI 1.06 to 1.17), respectively. When multivariable models were stratified according to sex, the risk of depression among males (aHR 1.11; 95% CI 1.06 to 1.16) and the risk of anxiety among both males (aHR 1.15; 95% CI 1.06 to 1.25) and females (aHR 1.09; 95% CI 1.02 to 1.16) remained significant for those with CRC compared to controls. Evaluating the risk of depression and anxiety by age at CRC diagnosis resulted in similar findings for the aCRC group. For instance, the multivariable models show an increased risk of depression (aHR 1.03; 95% CI 1.00 to 1.07) and anxiety (aHR 1.11; 95% CI 1.05 to 1.18) for individuals with aCRC compared to cancer-free controls. The findings particularly diverged for individuals with yCRC, where the multivariable models indicated that only males with yCRC, compared to male controls, had an increased risk for incident depression (aHR 1.17; 95% CI 1.03 to 1.33).

### 3.3. Depression and Anxiety According to Phases of CRC Care

When analyzing phases of CRC care at the time of incident depression or anxiety, we found that 17.5% of incident depression and 19.8% of incident anxiety occurred during the initial phase (i.e., 12 months after CRC). Incident cases of depression and anxiety were most commonly diagnosed in the continuing phase (i.e., between initial and end-of-life) at 61.7% and 63.6%, respectively. Finally, during the end-of-life phase (i.e., 12 months before cancer death), 13.3% of new cases of depression and 12.0% of new cases of anxiety occurred.

## 4. Discussion

In our retrospective longitudinal cohort study that used population-based administrative health data to identify 54,634 individuals with CRC over a 30-year period, we found that individuals with yCRC have a 41% increased risk for depression in comparison to individuals with aCRC (aHR 1.41; 95% CI 1.25 to 1.60). Stratifying the primary analysis by sex indicated that this increased risk is a result of males with yCRC having a 76% higher risk of depression compared to males with aCRC (aHR 1.76; 95% CI 1.48 to 2.10), an association that was non-significant within the female stratum (aHR 1.17; 95% CI 0.99 to 1.38). Our secondary analysis, which aimed to assess the risk of depression and anxiety in individuals with CRC, yCRC and aCRC in comparison to cancer-free controls, demonstrated that the overall risk of depression and anxiety was modestly increased for individuals with CRC and aCRC. Findings of the secondary analysis differed for individuals with yCRC, as the overall risk of depression and anxiety in comparison to cancer-free controls was non-significant; however, stratification by sex indicated that males with yCRC had a 17% higher risk of depression (aHR 1.17; 95% CI 1.03 to 1.33). Altogether, the increased incidence of mental health disorders for individuals with CRC raises awareness on mental health impacts of the disease and the importance of ensuring care and support for patients.

Similar to the increased risk of depression (aHR 1.03; 95% CI 1.00 to 1.06) and anxiety (aHR 1.11; 95% CI 1.06 to 1.17) among individuals with CRC in comparison to controls reported in our study, Lloyd et al. [6] found that individuals with CRC were at an increased risk for both depression and anxiety after diagnosis, with the highest risk occurring between 0–2 years (anxiety—aHR 2.84; 95% CI 2.55 to 3.17; depression—aHR 2.62; 95% CI 2.35 to 2.93). Furthermore, Sun et al. [5] also found that individuals with CRC had a higher risk of developing depressive disorders (aHR 1.54; 95% CI 1.39 to 1.71) and anxiety disorders (aHR 3.81; 95% CI 3.59 to 4.04) compared to general population controls. Many factors could contribute to these elevated risk estimates for depression and anxiety compared to our present study, among which are differing data sources and study populations. It is imperative to note that our study utilized validated case definitions for depression and anxiety using ICD-9 and ICD-10 codes, which previous publications did not integrate. Validated case definitions are the gold standard for reducing bias in exposure and outcome ascertainment, rendering this a major strength of our results [27]. Moreover, a unique finding of our study is those with yCRC had an increased risk of depression as compared to aCRC (aHR 1.41; 95% CI 1.25 to 1.60). In terms of anxiety, there was a non-significant difference in risk between the yCRC and aCRC cohort (aHR 1.05; 95% CI 0.86 to 1.28). Our comparison of yCRC and aCRC is an important addition to the literature and is supported by previous studies that show an inverse relationship between depression and age [28]. This is indicative of the need for person-centered mental health services for those who have cancer, especially younger individuals, as they are at an increased risk of depression and anxiety [29]. The phase of mental health issue occurrence should also be considered when developing resources, as there are undoubtedly unique needs in the initial, continuing, and end-of-life phases in the trajectory of CRC care.

By disaggregating the analyses in our study according to sex, we identified that males with yCRC experience a greater risk of incident depression compared to males with aCRC as well as in comparison to cancer-free controls. This contrasts with evidence from the general population, where females have a 2-times higher prevalence and an increased cumulative incidence of depression compared to males [30,31]. Explanations for these differences in the general population include men’s adherence to traditional masculine norms, which is associated with decreased help-seeking behaviours and the expression of atypical externalizing symptoms (e.g., anger, irritability, and impulsivity) of depression [32,33,34]. It is plausible that some barriers may be overcome in the context of CRC, where men have gained access to the health care system and clinicians, thereby helping to facilitate the diagnosis of mental health disorders. Additionally, our results are similar to the findings from Sun et al. [5], where male (aHR 3.19; 95% CI 2.98 to 3.42) and female (aHR 2.86; 95% CI 2.65 to 3.08) patients with CRC, as compared to controls, presented with a higher risk of developing mood disorders, with the highest risk observed among males. This contrasts with observations in various other cancer types, for example lung cancer, where stratified analyses indicate the risk of depression was higher among females (aHR 1.88; 95% CI 1.40 to 2.54) than males (aHR 1.49; 95% CI 1.18 to 1.89) [35]. These dissimilar results prompt the need for future research to identify why a higher risk of depression is occurring in males with CRC, and notably those with yCRC.

The study strengths and limitations warrant discussion. A strength of our study linkage of data from Population Data BC and the BC Cancer Registry, creating a source population spanning over 30 years. The BC Cancer Registry is reviewed annually for quality, completeness, and accuracy by the North American Association of Central Cancer Registries. Nonetheless, this is a retrospective study utilizing administrative data, which has inherent limitations with respect to the accuracy of the data and changes in practices (e.g., coding) over the study period. For example, we were limited to including information on stage in sensitivity analyses over the time period, 1 January 2010 to 31 December 2017, corresponding to the beginning of systematic collection of information on stage in the BC Cancer Registry. As described in the Methods, to date, administrative databases in BC do not yet capture information on the social construct of gender and as such, we are not able to incorporate this into our analysis.

Overall, our results suggest that a diagnosis of CRC at any age is associated with incident mental health disorders, and a particularly increased risk of depression is observed among males diagnosed with yCRC. Clinical attention to the onset of depression and anxiety among both female and male patients with CRC is essential throughout the entire trajectory of CRC care.

## Figures and Tables

**Table 1:** Characteristics of individuals with colorectal cancer (CRC), young-onset colorectal cancer (yCRC; <50 years), average-age-onset colorectal cancer (aCRC; ≥50 years), and their respective controls.

Characteristic	CRC (*n* = 54,634)	Controls (*n* = 546,340)	yCRC (*n* = 4223)	Controls (*n* = 42,230)	aCRC (*n* = 50,411)	Controls (*n* = 504,110)
Age, mean (SD)	67.9 (11.9)	67.9 (11.9)	43.2 (6.0)	43.1 (6.0)	70.0 (9.8)	70.0 (9.8)
Female, *n* (%)	25,402 (46.5)	254,020 (46.5)	2091 (49.5)	20,910 (49.5)	23,311 (46.2)	233,110 (46.2)
Charlson–Romano comorbidity index, mean (SD)	0.91 (2.12)	0.14 (0.60)	0.81 (2.06)	0.04 (0.35)	0.92 (2.13)	0.14 (0.62)
Neighbourhood income quintile, *n* (%)						
Quintile 1	11,301 (20.7)	116,633 (21.4)	774 (18.3)	8723 (20.7)	10,527 (20.9)	107,910 (21.4)
Quintile 2	10,325 (18.9)	108,446 (19.9)	794 (18.8)	8521 (20.2)	9531 (18.9)	99,925 (19.8)
Quintile 3	13,166 (24.1)	118,427 (21.7)	1024 (24.3)	8645 (20.5)	12,142 (24.1)	109,782 (21.8)
Quintile 4	9850 (18.0)	100,190 (18.3)	828 (19.6)	8410 (19.9)	9022 (17.9)	91,780 (18.2)
Quintile 5	9992 (18.3)	102,644 (18.8)	803 (19.0)	7931 (18.8)	9189 (18.2)	94,713 (18.8)
Residence, *n* (%)						
Urban	46,510 (85.1)	468,596 (85.8)	3691 (87.4)	37,276 (88.3)	42,819 (84.9)	431,320 (85.6)
Rural	8124 (14.9)	77,744 (14.2)	532 (12.6)	4954 (11.7)	7592 (15.1)	72,790 (14.4)
Health care use, mean (SD)						
Number of outpatient visits	14.1 (12.6)	10.0 (11.4)	10.2 (9.8)	9.3 (12.5)	14.4 (12.7)	10.1 (11.3)
Number of inpatient visits	0.8 (1.1)	0.3 (0.8)	0.7 (1.0)	0.2 (0.7)	0.9 (1.1)	0.3 (0.8)

Descriptive statistics were determined for the year prior to index date (the 1-year leading up to and including index date). Abbreviations: SD—standard deviation.

**Table 2:** Risk of depression and anxiety in individuals diagnosed with young-onset colorectal cancer (yCRC; <50 years) compared to those with average-age-onset colorectal cancer (aCRC; ≥50 years) (1985–2017).

	Depression HR (95% CI)	Anxiety HR (95% CI)
**1: Unadjusted model**		
yCRC (vs. aCRC)	1.15 (1.05, 1.25)	1.30 (1.12, 1.50)
**2: Multivariable model ^a,b^**		
yCRC (vs. aCRC)	1.41 (1.25, 1.60)	1.05 (0.86, 1.28)
**3: Unadjusted models stratified by sex ^c^**		
Males, yCRC (vs. aCRC)	1.26 (1.11, 1.43)	1.34 (1.06, 1.70)
Females, yCRC (vs. aCRC)	1.03 (0.91, 1.17)	1.23 (1.02, 1.49)
**4: Multivariable models stratified by sex ^c^**		
Males, yCRC (vs. aCRC)	1.76 (1.48, 2.10)	1.00 (0.72, 1.38)
Females, yCRC (vs. aCRC)	1.17 (0.99, 1.38)	1.07 (0.83, 1.38)

^a^ Adjusted for age, sex, Charlson–Romano comorbidity index, neighbourhood income quintile, residence, number of outpatient visits, and number of inpatient visits. ^b^ See Supplementary Table S1 for full multivariable model. ^c^ Adjusted for age, Charlson–Romano comorbidity index, neighbourhood income quintile, residence, number of outpatient visits, and number of inpatient visits.

**Table 3:** Risk of depression and anxiety in individuals diagnosed with young-onset colorectal cancer (yCRC; <50 years) compared to those with average-age-onset colorectal cancer (aCRC; ≥50 years) adjusting for stage of CRC (2010–2016).

	Depression HR (95% CI)	Anxiety HR (95% CI)
**1: Unadjusted model**		
yCRC (vs. aCRC)	1.15 (1.05, 1.25)	1.30 (1.12, 1.50)
**2: Multivariable model ^a,b^**		
yCRC (vs. aCRC)	1.56 (1.13, 2.14)	1.17 (0.79, 1.75)
**3: Unadjusted models stratified by sex ^c^**		
Males, yCRC (vs. aCRC)	1.26 (1.11, 1.43)	1.34 (1.06, 1.70)
Females, yCRC (vs. aCRC)	1.03 (0.91, 1.17)	1.23 (1.01, 1.49)
**4: Multivariable models stratified by sex ^c^**		
Males, yCRC (vs. aCRC)	1.60 (1.02, 2.52)	0.93 (0.47, 1.83)
Females, yCRC (vs. aCRC)	1.49 (0.93, 2.37)	1.34 (0.81, 2.22)

^a^ Adjusted for cancer stage (0/1, 2, 3, 4), age, sex, Charlson–Romano comorbidity index, neighbourhood income quintile, residence, number of outpatient visits, and number of inpatient visits. ^b^ See Supplementary Table S2 for full multivariable model. ^c^ Adjusted for cancer stage (0/1, 2, 3, 4), age, Charlson–Romano comorbidity index, neighbourhood income quintile, residence, number of outpatient visits, and number of inpatient visits.

**Table 4:** Unadjusted and adjusted hazard ratios of depression and anxiety in individuals diagnosed with colorectal cancer (CRC; ≥18 years), young-onset colorectal cancer (yCRC; <50 years), average-age-onset colorectal cancer (aCRC; ≥50 years) as compared to their respective controls (1985–2017).

	CRC HR (95% CI)		yCRC HR (95% CI)		aCRC HR (95% CI)	
1: Depression	Unadjusted	Adjusted	Unadjusted	Adjusted	Unadjusted	Adjusted
All ^a,b^	1.12 (1.09, 1.15)	1.03 (1.00, 1.06)	1.02 (0.94, 1.12)	1.00 (0.92, 1.10)	1.13 (1.10, 1.17)	1.03 (1.00, 1.07)
Females ^c^	1.04 (1.00, 1.08)	0.97 (0.93, 1.01)	0.88 (0.78, 1.00)	0.88 (0.78, 1.00)	1.06 (1.01, 1.10)	0.98 (0.94, 1.03)
Males ^c^	1.22 (1.17, 1.27)	1.11 (1.06, 1.16)	1.22 (1.07, 1.38)	1.17 (1.03, 1.33)	1.22 (1.17, 1.27)	1.10 (1.05, 1.15)
**2: Anxiety**						
All ^a,b^	1.19 (1.13, 1.25)	1.11 (1.06, 1.17)	1.08 (0.94, 1.25)	1.10 (0.95, 1.27)	1.20 (1.14, 1.27)	1.11 (1.05, 1.18)
Females ^c^	1.15 (1.08, 1.23)	1.09 (1.02, 1.16)	1.08 (0.90, 1.29)	1.10 (0.91, 1.33)	1.17 (1.09, 1.25)	1.09 (1.02, 1.17)
Males ^c^	1.23 (1.14, 1.33)	1.15 (1.06, 1.25)	1.10 (0.88, 1.38)	1.10 (0.88, 1.39)	1.25 (1.15, 1.36)	1.16 (1.06, 1.26)

^a^ Adjusted for age, sex, Charlson–Romano comorbidity index, neighbourhood income quintile, residence, number of outpatient visits, and number of inpatient visits. ^b^ See Supplementary Table S3 for full multivariable models. ^c^ Adjusted for age, Charlson–Romano comorbidity index, neighbourhood income quintile, residence, number of outpatient visits, and number of inpatient visits.

## Data Availability

The data that support the findings of this study are available from Population Data BC [https://www.popdata.bc.ca/] (accessed on 15 October 2021) but restrictions apply to the availability of these data, which were used under license for the current study, and so are not publicly available. Data are available from Population Data BC through a data access request [dataaccess@popdata.bc.ca].

## Supplementary Materials

The following supporting information can be downloaded at: https://www.mdpi.com/article/10.3390/curroncol29050249/s1, Table S1: Risk of depression and anxiety in individuals diagnosed with young-onset colorectal cancer (yCRC; <50 years) compared to those with average age-onset colorectal cancer (aCRC; ≥50 years) (1985–2017); Table S2: Risk of depression and anxiety in individuals diagnosed with young-onset colorectal cancer (yCRC; <50 years) compared to those with average age-onset colorectal cancer (aCRC; ≥50 years) adjusting for stage of CRC (2010–2016). Table S3: Unadjusted and adjusted hazard ratios of depression and anxiety in individuals diagnosed with colorectal cancer (CRC; ≥18 years), young-onset colorectal cancer (yCRC; <50 years), average age-onset colorectal cancer (aCRC; ≥50 years) as compared to their respective controls (1985–2017).
