# Supplementary material for: Risk of Anxiety and Depression after Diagnosis of Young-Onset Colorectal Cancer: A Population-Based Cohort Study
Source: Curr Oncol. 2022 Apr 27;29(5):3072–81. doi: 10.3390/curroncol29050249 (PMC9140150; doi:10.3390/curroncol29050249)
Supplement: Supplementary file 1 [file curroncol-29-00249-s001.zip › curroncol-1673731-supplementary.pdf]

Supplementary Material

Supplementary Table 1. Risk of depression and anxiety in individuals diagnosed with young-onset colorectal cancer (yCRC; <50 years) compared to those with average age-onset colorectal cancer (aCRC; ≥50 years) (1985-2017)

	**Depression HR (95% CI)**	**Anxiety HR (95% CI)**
**1: Unadjusted model**		
yCRC (vs. aCRC)	1.15 (1.05, 1.25)	1.30 (1.12, 1.50)
**2: Multivariable model**		
yCRC (vs. aCRC)	1.41 (1.25, 1.60)	1.05 (0.86, 1.28)
Age	1.01 (1.00, 1.01)	0.99 (0.98, 1.00)
Sex (female vs. male)	1.23 (1.16, 1.30)	1.63 (1.48, 1.80)
Neighbourhood income quintile	0.98 (0.96, 1.00)	0.97 (0.94, 1.00)
Charlson-Romano comorbidity index	1.01 (0.99, 1.03)	0.97 (0.94, 1.01)
Residence (rural vs. urban)	0.85 (0.79, 0.93)	0.92 (0.79, 1.06)
Number of outpatient visits	1.02 (1.02, 1.02)	1.02 (1.02, 1.02)
Number of inpatient visits	0.96 (0.93, 0.99)	0.97 (0.92, 1.02)

Supplementary Table 2. Risk of depression and anxiety in individuals diagnosed with young-onset colorectal cancer (yCRC; <50 years) compared to those with average age-onset colorectal cancer (aCRC; ≥50 years) adjusting for stage of CRC (2010-2016)

	**Depression HR (95% CI)**	**Anxiety HR (95% CI)**
**1: Unadjusted model**		
yCRC (vs. aCRC)	1.15 (1.05, 1.25)	1.30 (1.12, 1.50)
**2: Multivariable model**		
yCRC (vs. aCRC)	1.56 (1.13, 2.14)	1.17 (0.79, 1.75)
CRC stage 2 (vs. stage 1)	1.56 (1.23, 1.97)	1.15 (0.85, 1.55)
CRC stage 3 (vs. stage 1)	1.80 (1.44, 2.24)	1.45 (1.10, 1.92)
CRC stage 4 (vs. stage 1)	2.30 (1.75, 3.02)	1.64 (1.14, 2.36)
Age	0.99 (0.98, 1.00)	0.98 (0.96, 0.99)
Sex (female vs. male)	1.29 (1.11, 1.51)	1.96 (1.59, 2.42)
Neighbourhood income quintile	0.97 (0.92, 1.02)	1.00 (0.93, 1.08)
Charlson-Romano comorbidity index	0.99 (0.94, 1.05)	1.00 (0.93, 1.07)
Residence (rural vs. urban)	0.87 (0.69, 1.11)	0.92 (0.67, 1.26)
Number of outpatient visits	1.02 (1.01, 1.02)	1.02 (1.01, 1.02)
Number of inpatient visits	0.95 (0.86, 1.05)	1.00 (0.89, 1.11)

Supplementary Table 3. Unadjusted and adjusted hazard ratios of depression and anxiety in individuals diagnosed with colorectal cancer (CRC; ≥ 18 years), young-onset colorectal cancer (yCRC; <50 years), average age-onset colorectal cancer (aCRC; ≥50 years) as compared to their respective controls (1985-2017)

	CRC<br>HR (95% CI)	yCRC<br>HR (95% CI)	aCRC<br>HR (95% CI)
**1: Depression**			
**Unadjusted model**			
CRC (vs. control)	1.12 (1.09, 1.15)	1.02 (0.94, 1.12)	1.13 (1.10, 1.17)
**Adjusted model**			
CRC (vs. control)	1.03 (1.00, 1.06)	1.00 (0.92, 1.10)	1.03 (1.00, 1.07)
Age	1.00 (1.00, 1.01)	0.99 (0.98, 0.99)	1.02 (1.02, 1.02)
Sex (female vs. male)	1.43 (1.41, 1.45)	1.42 (1.36, 1.48)	1.38 (1.36, 1.40)
Neighbourhood income quintile	0.98 (0.98, 0.99)	0.95 (0.94, 0.97)	0.99 (0.98, 0.99)
Charlson-Romano comorbidity index	1.01 (1.00, 1.02)	0.99 (0.95, 1.03)	1.02 (1.00, 1.03)
Residence (rural vs. urban)	0.97 (0.95, 0.99)	1.03 (0.96, 1.10)	0.98 (0.96, 1.00)
Number of outpatient visits	1.02 (1.02, 1.02)	1.02 (1.02, 1.02)	1.02 (1.02, 1.02)
Number of inpatient visits	1.02 (1.01, 1.03)	1.01 (0.98, 1.04)	1.02 (1.01, 1.03)
**2: Anxiety**			
**Unadjusted model**			
CRC (vs. control)	1.19 (1.13, 1.25)	1.08 (0.94, 1.25)	1.20 (1.14, 1.27)
**Adjusted model**			
CRC (vs. control)	1.11 (1.06, 1.17)	1.10 (0.95, 1.27)	1.11 (1.05, 1.18)
Age	0.99 (0.99, 0.99)	0.99 (0.98, 0.99)	1.00 (1.00, 1.00)
Sex (female vs. male)	1.72 (1.68, 1.77)	1.54 (1.43, 1.65)	1.73 (1.68, 1.78)
Neighbourhood income quintile	1.01 (1.00, 1.02)	0.99 (0.96, 1.02)	1.01 (1.00, 1.02)
Charlson-Romano comorbidity index	0.93 (0.91, 0.96)	0.91 (0.84, 0.98)	0.94 (0.91, 0.96)
Residence (rural vs. urban)	1.06 (1.02, 1.10)	1.02 (0.91, 1.13)	1.07 (1.03, 1.11)
Number of outpatient visits	1.02 (1.02, 1.02)	1.02 (1.02, 1.02)	1.02 (1.02, 1.02)
Number of inpatient visits	1.02 (1.01, 1.04)	1.05 (1.01, 1.09)	1.02 (1.00, 1.04)
